# Themis regulates metabolic signaling and effector functions in CD4^+^ T cells by controlling NFAT nuclear translocation

**DOI:** 10.1038/s41423-020-00578-4

**Published:** 2020-11-11

**Authors:** Mukul Prasad, Joanna Brzostek, Namrata Gautam, Renu Balyan, Vasily Rybakin, Nicholas R. J. Gascoigne

**Affiliations:** 1 Translational Immunology Programme, Yong Loo Lin School of Medicine, National University of Singapore, 5 Science Drive 2, Singapore, 117545 Singapore; 2 Department of Microbiology and Immunology, Yong Loo Lin School of Medicine, National University of Singapore, 5 Science Drive 2, Singapore, 117545 Singapore; 3 Present Address: H. Lee Moffitt Cancer Center and Research Institute, Tampa, FL USA; 4 Present Address: Torque Therapeutics, Cambridge, MA USA

**Keywords:** Immunometabolism, NFAT nuclear translocation, Insulin receptor, mTOR, Mitochondria, CD4-positive T cells, Insulin signalling, Signal transduction, Protein translocation

## Abstract

Themis is a T cell lineage-specific molecule that is involved in TCR signal transduction. The effects of germline Themis deletion on peripheral CD4^+^ T cell function have not been described before. In this study, we found that Themis-deficient CD4^+^ T cells had poor proliferative responses, reduced cytokine production in vitro and weaker inflammatory potential, as measured by their ability to cause colitis in vivo. Resting T cells are quiescent, whereas activated T cells have high metabolic demands. Fulfillment of these metabolic demands depends upon nutrient availability and upregulation of nutrient intake channels after efficient TCR signal transduction, which leads to metabolic reprogramming in T cells. We tested whether defects in effector functions were caused by impaired metabolic shifts in Themis-deficient CD4^+^ T cells due to inefficient TCR signal transduction, in turn caused by the lack of Themis. We found that upon TCR stimulation, Themis-deficient CD4^+^ T cells were unable to upregulate the expression of insulin receptor (IR), glucose transporter (GLUT1), the neutral amino acid transporter CD98 and the mTOR pathway, as measured by c-Myc and pS6 expression. Mitochondrial analysis of activated Themis-deficient CD4^+^ T cells showed more oxidative phosphorylation (OXPHOS) than aerobic glycolysis, indicating defective metabolic reprogramming. Furthermore, we found reduced NFAT translocation in Themis-deficient CD4^+^ T cells upon TCR stimulation. Using previously reported ChIP-seq and RNA-seq data, we found that NFAT nuclear translocation controls *IR* gene expression. Together, our results describe an internal circuit between TCR signal transduction, NFAT nuclear translocation, and metabolic signaling in CD4^+^ T cells.

## Introduction

The main function of T cells is to recognize foreign antigens presented by MHC molecules and mount an immune response, which entails T cell activation, proliferation, and production of effector cytokines. All these effector functions are energy demanding processes and are coupled with changes in cellular metabolism to access the right nutrients and meet new metabolic demands.^1^ Glucose is a major source of energy for T cells, which take up glucose using the glucose transporter GLUT1^2^ upon insulin receptor (IR) signaling,^3^ subjecting it to glycolysis for energy production. Recent studies in rodents have shown that IR-deficient T cells have reduced T cell effector functions, demonstrated by their inability to mount an effective immune response in infection models and to initiate inflammation in experimental autoimmune encephalitis and colitis animal models.^4,5^

Glycolysis ends with the production of pyruvate, which can have two fates: it can either be broken down into lactate via aerobic glycolysis or transported to mitochondria, used for the tricarboxylic acid (TCA) or Krebs cycle and ultimately converted to ATP via oxidative phosphorylation (OXPHOS).^6,7^ Resting T cells are quiescent and do not have high metabolic demands. Upon antigen recognition, T cells become activated and switch from OXPHOS to aerobic glycolysis, which consumes more glucose to generate the same amount of ATP as OXPHOS but does so more rapidly. This requires increased glucose uptake and therefore more glucose transporters.^6,7^ TCR signaling upon antigen recognition leads to activation of the kinase mTOR,^8^ which is required to induce the expression of many genes involved in metabolic pathways, such as GLUT1,^9^ and activate other downstream enzymes, such as c-Myc.^10^ c-Myc is important for inducing the expression of enzymes involved in glycolysis, glutaminolysis and other metabolic pathways that activated T cells require for proliferation and effector functions.^11^

Although it has been shown that upon T cell activation, T cells shift from OXPHOS to aerobic glycolysis, this does not mean that OXPHOS is completely stopped. Along with glucose, glutamine is also taken into T cells upon activation.^12^ Glutamine can be converted to glutamate in the mitochondria and can further be converted to α-ketoglutarate, one of the intermediaries for the TCA cycle, thus ensuring that the OXPHOS process does not come to a halt.^11^ T cell activation and effector function can be maintained by the mitochondria alone. The reactive oxygen species (ROS) produced by mitochondria during OXPHOS are required for NFAT activation.^13^ NFAT is one of the key transcription factors that translocates to the nucleus soon after TCR signaling to allow transcription of various genes, including IL-2.^14^

Themis is a T-cell lineage-specific molecule with a critical role in thymocyte selection.^15–19^ Themis constitutively interacts with the adaptor protein Grb2 and with the phosphatase Shp1, affecting its activity,^15–21^ but the precise molecular function of Themis in the regulation of signaling pathways in T cells remains controversial. Themis has been proposed to act as a negative regulator of TCR signaling, with impaired thymocyte selection in mice with Themis germline knockout (KO) resulting from enhanced negative selection.^21,22^ However, an alternative model proposes that Themis is a positive regulator of TCR signaling, attributing compromised thymocyte development to reduced positive selection.^23^ One of the early papers describing Themis suggested that the main effect of Themis during thymocyte development is an effect on the regulation of metabolism rather than on TCR signaling.^17^ Themis germline KO mice have severely reduced numbers of CD4^+^ T cells and increased percentages of Tregs and memory-phenotype CD44^hi^ CD4^+^ T cells.^15–18^ However, post-selection Themis deletion does not alter CD4^+^ T cell numbers and has a minor effect on their phenotype.^24^ The role of Themis in peripheral CD4^+^ T cells is still poorly understood.

The modulation of T cell effector functions by changes in TCR signaling strength has been studied extensively, and several studies have reported how the metabolic landscape of a T cell changes with differing TCR signal strength. The strength of the TCR stimulus determines the frequency of cells that express c-Myc,^25^ and a recent paper showed a circuit linking TCR signal strength and the amount of acetyl-CoA modulated by Akt.^26^ However, there is little information on how intrinsic variations within a T cell that lead to signal transduction differences can affect its metabolic profile. Themis-deficient mature CD4^+^ T cells have been shown to respond poorly to TCR stimulation,^15^ but the molecular basis of this defect is unknown. To understand the molecular basis, we investigated metabolic signaling in Themis-deficient CD4^+^ T cells and their effector functions. Our results show that a lack of efficient TCR signal transduction due to the absence of Themis leads to decreased effector functions in vitro and in vivo, reduced IR signaling and nutrient uptake, defective metabolic reprogramming of mitochondria and defective mTOR activation. Ultimately, we show that these defects in metabolic signaling are regulated via NFAT translocation to the nucleus and its control of IR expression.

## Results

### Themis deficiency reduces CD4^+^ T cell function in vitro

**Fig. 1:**
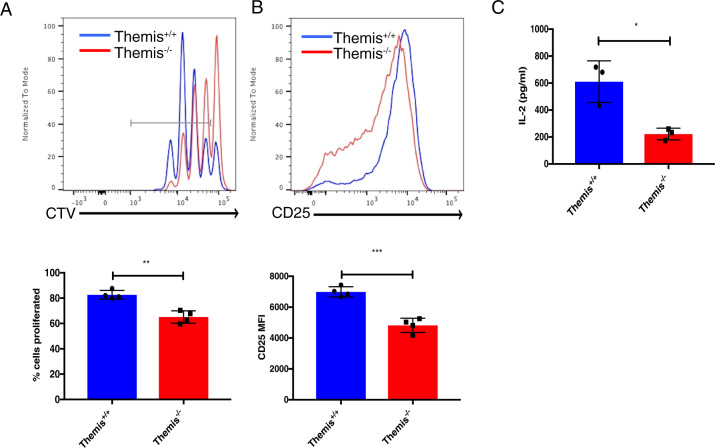
CD4^+^ T cells from *Themis*^*-/-*^ mice have defective effector functions in vitro. **A** Histogram and bar graph showing the proliferation of CD4^+^ T cells from *Themis*^*-/-*^ and *Themis*^*+/+*^ mice in response to anti-CD3/CD28 stimulation for 72 h. **B** Histogram and bar graph showing CD25 expression on CD4^+^ T cells from *Themis*^*-/-*^ and *Themis*^*+/+*^ mice in response to anti-CD3/CD28 stimulation for 24 h. **C** IL-2 production by CD4^+^ T cells from *Themis*^*-/-*^ and *Themis*^*+/+*^ mice in response to anti-CD3/CD28 stimulation for 24 h. Data are representative of three individual experiments with *n* = 3–4 technical replicates per genotype per experiment. *p < 0.5, **p < 0.01, ***p < 0.001 as determined by Student’s *t* test. All error bars represent SDs

Themis germline KO mice present a lymphopenic phenotype in the periphery, with a significant decrease in the proportion of CD4^+^ T cells.^15–18^ The effect of Themis deletion on CD4^+^ T cell function in the periphery has not been thoroughly examined. We therefore tested two of the most fundamental T cell effector functions: proliferative responses and cytokine production. Naïve CD4^+^ Foxp3^-^ CD44^lo^ T cells sorted from *Themis*^*+/+*^ and *Themis*^*-/-*^ mice were stimulated with anti-CD3/CD28. The gating strategy used for sorting is shown in Supplementary Fig. 1. Cell trace violet (CTV) labeling was used to assess in vitro proliferation after 72 h of activation. *Themis*^*-/-*^ CD4^+^ T cells proliferated significantly less than *Themis*^*+/+*^ CD4^+^ T cells (Fig. 1A). An IL-2 ELISA was performed to assess cytokine production after 24 h of activation, and CD25 expression was evaluated to check the activation state of these cells. *Themis*^*-/-*^ CD4^+^ T cells had lower CD25 cell surface expression and reduced production of IL-2 (Fig. 1B, C). These results showing a deficit in cytokine production and proliferation point toward a decrease in effector functions in Themis-deficient CD4^+^ T cells.

### Themis deficiency affects CD4^+^ T cell function in vivo

**Fig. 2:**
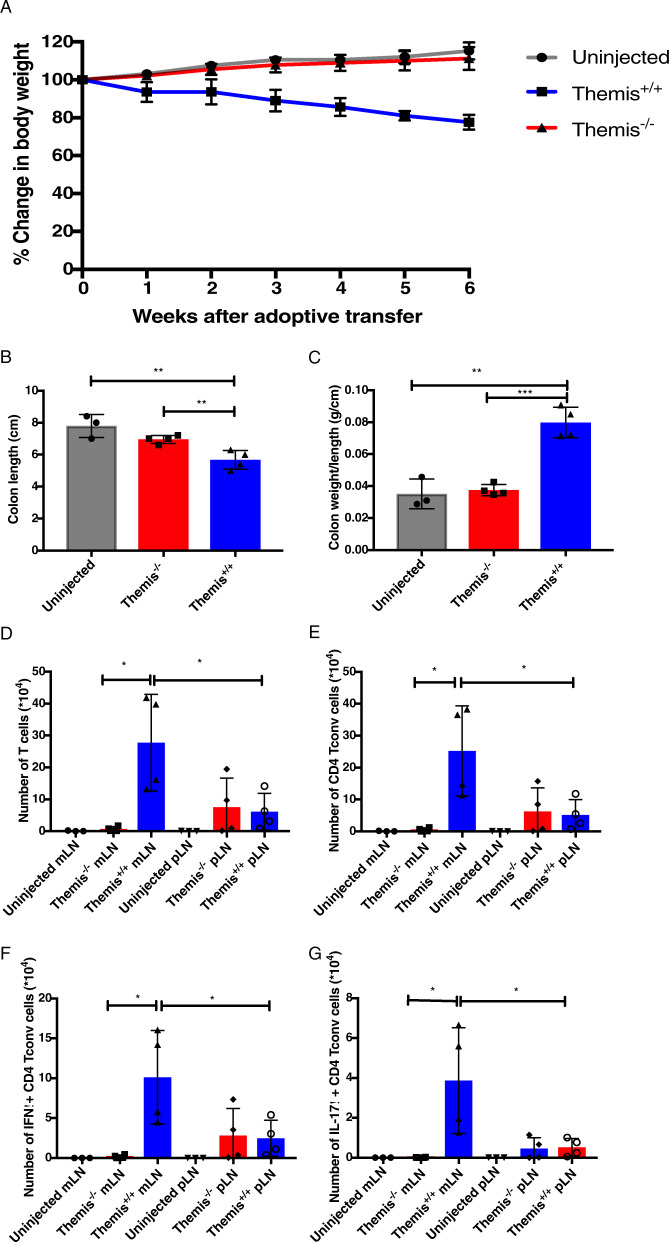
CD4^+^ T cells from *Themis*^*-/-*^ mice have defective effector functions in vivo. Naïve sorted CD4^+^ T cells from *Themis*^*-/-*^ and *Themis*^*+/+*^ mice were adoptively transferred into *Rag1*^*-/-*^ hosts. **A** Percent change in body weight of *Rag1*^*-/-*^ hosts after adoptive transfer. Error bars represent SEMs. Colon (**B**) length and (**C**) weight/length ratio of *Rag1*^*-/-*^ hosts at 6 weeks after adoptive transfer. Absolute numbers of (**D**) total T cells, (**E**) total CD4^+^, (**G**) IFNγ^+^CD4^+^Foxp3^-^ and (**H**) IL-17α^+^CD4^+^Foxp3^-^ T cells in the peripheral and mesenteric lymph nodes of *Rag1*^*-/-*^ hosts at 6 weeks after adoptive transfer. Data are representative of two individual experiments with *n* = 3–4 biological replicates per group per experiment. *p < 0.5, **p < 0.01, ***p < 0.001 as determined by Student’s *t* test. All error bars represent SDs except those in 2(**a**), which represent the standard error mean

Next, we investigated the in vivo effector functions of Themis-deficient CD4^+^ T cells using an adoptive transfer model of colitis.^27^ This model tests for the ability of CD4^+^ T cells to induce colonic inflammation by proliferation and cytokine production in response to the gut flora in *Rag*-deficient hosts. This colonic inflammation leads to a decrease in the length and thickness of the colon tissue, leading to malabsorption of nutrients, weight loss, and ultimately death.^27^ Naïve CD4^+^ Foxp3^-^ T cells sorted from *Themis*^*+/+*^ and *Themis*^*-/-*^ mice were injected intraperitoneally into *Rag1*^*-/-*^ mice. *Rag1*^*-/-*^ mice that received *Themis*^*-/-*^ CD4^+^ Foxp3^-^ T cells were resistant to developing colitis compared to the *Rag1*^*-/-*^ mice that received *Themis*^*+/+*^ CD4^+^ Foxp3^-^ T cells, as measured by increased body weight (Fig. 2A). Histopathological characterization of the colon showed decreased inflammation in mice that received *Themis*^*-/-*^ cells compared to mice that received *Themis*^*+/+*^ cells. There were no changes in colon length and weight in the mice that received *Themis*^*-/-*^ cells compared to uninjected mice and a decreased colon weight/length ratio compared to that of the mice that received *Themis*^*+/+*^ cells (Fig. 2B, C). Further investigation of gut-draining mesenteric lymph nodes revealed significantly reduced accumulation and proliferation of total T cells and CD4^+^ Foxp3^-^ T cells in the mice that received *Themis*^*-/-*^ CD4^+^ Foxp3^-^ T cells compared to the mice that received *Themis*^*+/+*^ CD4^+^ Foxp3^-^ T cells (Fig. 2D, E). *Themis*^*-/-*^ CD4^+^ Foxp3^-^ T cells showed a significant reduction in the proportion of T_H_1-polarized (IFNγ-producing) (Fig. 2F) and T_H_17-polarized (IL-17α-producing) cells (Fig. 2G) after restimulation with anti-CD3/CD28 compared to that of *Themis*^*+/+*^ CD4^+^ Foxp3^-^ T cells. Peripheral lymph nodes were analyzed as controls and revealed no significant changes in the accumulation and proliferation of either total T cells or CD4^+^ Foxp3^-^ T cells, nor in the proportions of T_H_1-polarized and T_H_17-polarized cells, between the mice that received *Themis*^*-/-*^ CD4^+^ Foxp3^-^ T cells and the mice that received *Themis*^*+/+*^ CD4^+^ Foxp3^-^ T cells (Fig. 2D–G), pointing toward the reduced capability of *Themis*^*-/-*^ CD4^+^ Foxp3^-^ T cells to migrate to the gut. Taken together with the data from the in vitro functional assays (Fig. 1), these data show that Themis-deficient CD4^+^ T cells have a deficit in effector functions.

### Themis-deficient CD4^+^ T cells do not upregulate IR upon TCR stimulation

**Fig. 3:**
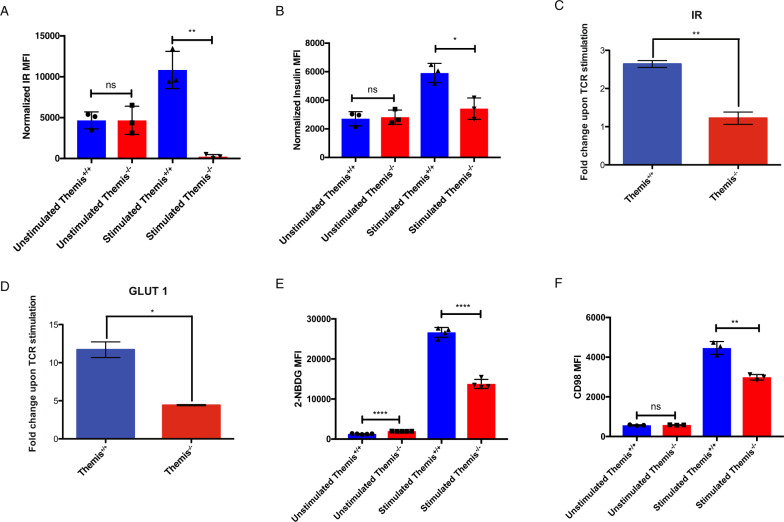
CD4^+^ T cells from *Themis*^*-/-*^ mice have defective IR signaling and nutrient uptake upon TCR stimulation. **A** IR expression on unstimulated and stimulated CD4^+^ T cells from *Themis*^*-/-*^ and *Themis*^*+/+*^ mice. **B** Ratio of insulin binding in stimulated and unstimulated CD4^+^ T cells from *Themis*^*-/-*^ and *Themis*^*+/+*^ mice. **C** *IR* mRNA expression changes in CD4^+^ T cells from *Themis*^*-/-*^ and *Themis*^*+/+*^ mice upon TCR stimulation. **D** *GLUT1* mRNA expression changes in CD4^+^ T cells upon TCR stimulation in *Themis*^*-/-*^ and *Themis*^*+/+*^ mice relative to unstimulated ex vivo CD4^+^ T cells. **E** Glucose uptake by unstimulated and stimulated CD4^+^ T cells from *Themis*^*-/-*^ and *Themis*^*+/+*^ mice. **F** CD98 expression on unstimulated and stimulated CD4^+^ T cells from *Themis*^*-/-*^ and *Themis*^*+/+*^ mice. Data representative of three individual experiments with *n* = 3–5 technical replicates per genotype. ^ns^not significant, *p < 0.5, **p < 0.01, ***p < 0.001, ****p < 0.0001 as determined by Student’s *t* test. All error bars represent SDs

Themis has been shown to be involved in TCR signaling^15,16,18^ and metabolic pathways in thymocytes,^17^ which led us to examine intersections between TCR and metabolic signaling pathways in CD4^+^ T cells. The insulin signaling pathway was of particular interest, as there are common binding partners between IR and Themis, such as Grb2,^3,20^ as well as common downstream signaling pathways, such as Erk and mTOR activation. This led us to hypothesize that there might be an interaction between the two pathways. We analyzed the expression of IR in naïve CD4^+^ Foxp3^-^ T cells sorted from *Themis*^*+/+*^ and *Themis*^*-/-*^ mice after stimulation with anti-CD3/CD28 for 72 h. The expression levels of IR were analyzed by flow cytometry (Fig. 3A). We observed that *Themis*^*-/-*^ CD4^+^ T cells did not upregulate IR relative to unstimulated ex vivo T cells upon TCR stimulation. To corroborate these IR expression data, we performed an insulin binding assay. The amount of fluorescent insulin bound to the T cells correlates with the surface expression of IR. The amount of insulin bound to stimulated *Themis*^*+/+*^ CD4^+^ T cells was much higher than that bound to stimulated *Themis*^-/-^ CD4^+^ T cells. In unstimulated T cells, the ratio of insulin binding between CD4^+^ T cells from *Themis*^*+/+*^ and *Themis*^*-/-*^ mice was close to 1, indicating that there was no difference in the amount of insulin bound to resting CD4^+^ T cells from *Themis*^*+/+*^ and *Themis*^*-/-*^ mice (Fig. 3B). To further validate these results, we measured *IR* mRNA expression by qPCR. To this end, naïve CD4^+^ T cells sorted from *Themis*^*+/+*^ and *Themis*^*-/-*^ mice were stimulated with anti-CD3/CD28 for 72 h. RNA from stimulated and unstimulated cells was converted to cDNA, which was used for gene expression analysis by qPCR. The qPCR results were consistent with the flow cytometry results, showing that CD4^+^ T cells from *Themis*^*-/-*^ mice were not able to upregulate *IR* upon TCR stimulation (Fig. 3C). This suggests that the downstream signaling of the IR pathway would be affected in *Themis*^-/-^ CD4^+^ T cells.

### Themis-deficient CD4^+^ T cells have defective nutrient uptake

IR and signaling mediated via IR are required to bring glucose transporter molecules such as GLUT1 to the cell surface to enable glucose uptake. To examine changes in *GLUT1* expression, we sorted naïve CD4^+^ Foxp3^-^ T cells from *Themis*^*+/+*^ and *Themis*^-/-^ mice and stimulated them with anti-CD3/CD28 for 72 h. We then analyzed *GLUT1* mRNA expression by qPCR. We observed that CD4^+^ T cells from *Themis*^*-/-*^ mice were unable to upregulate *GLUT1* expression upon TCR stimulation (Fig. 3D). This led us to hypothesize that there might be differences in glucose uptake by these T cells, which would be a functional readout for these cells’ metabolic activity upon TCR stimulation. We therefore performed a glucose uptake assay using a fluorescent glucose analog, 2-NBDG, on stimulated and unstimulated naïve sorted CD4^+^ Foxp3^-^ T cells from *Themis*^*+/+*^ and *Themis*^*-/-*^ mice. We observed that stimulated CD4^+^ T cells from *Themis*^*-/-*^ mice take up less glucose than CD4^+^ T cells from *Themis*^*+/+*^ mice (Fig. 3E). Next, we wanted to test whether the uptake or transport of other nutrients was also affected during the activation of *Themis*^*-/-*^ CD4^+^ T cells. We analyzed the surface expression of CD98, which is the transporter for large and neutral amino acids (valine, leucine, isoleucine, tryptophan and tyrosine).^28^ We observed that stimulated CD4^+^ T cells from *Themis*^*-/-*^ mice had reduced expression of CD98 in comparison to that of CD4^+^ T cells from *Themis*^*+/+*^ mice (Fig. 3F). These data show defective nutrient uptake by *Themis*^*-/-*^ CD4^+^ T cells, which might be a reason for the loss of their effector functions.

### Themis-deficient CD4^+^ T cells have defective mitochondrial function

**Fig. 4:**
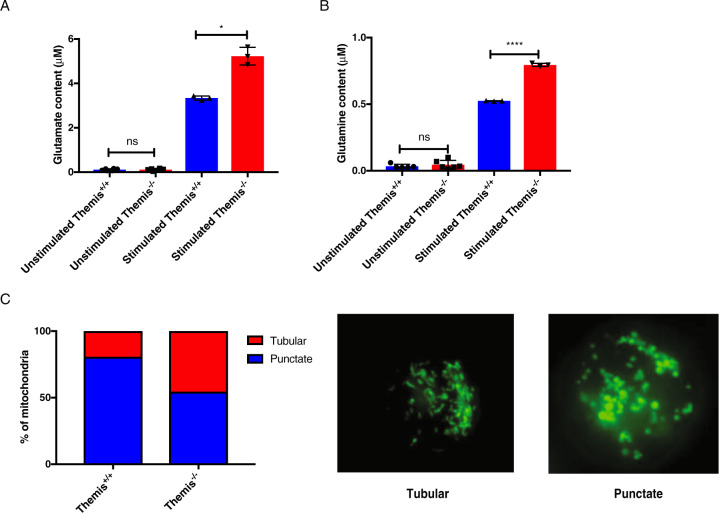
Mitochondrial dysfunction in *Themis*^*-/-*^ CD4^+^ T cells upon TCR stimulation. Intracellular (**A**) glutamate and (**B**) glutamine concentrations in unstimulated and stimulated CD4^+^ T cells from *Themis*^*-/-*^ and *Themis*^*+/+*^ mice. **C** Histological summary of mitochondrial morphology observed in stimulated CD4^+^ T cells from *Themis*^*-/-*^ and *Themis*^*+/+*^ mice and sample microscopic images of tubular and punctate mitochondria. Images of 50 cells per sample were analyzed for the morphology analysis. Data are representative of three individual experiments with 3–5 technical replicates per genotype per experiment. ^ns^not significant, *p < 0.5, ****p < 0.0001 as determined by Student’s *t* test. All error bars represent SDs

Under low-glucose conditions, such as those experienced by CD4^+^ T cells from *Themis*^*-/-*^ mice due to low-glucose uptake, T cells utilize glutamine^29^ and convert it to glutamate in the mitochondria. Glutamate is converted to α-ketoglutarate, one of the intermediaries for the TCA cycle, in a process called anapleurosis to fuel the TCA cycle.^11^ To test if this was occurring, we measured the glutamine and glutamate content in stimulated and unstimulated CD4^+^ T cells from *Themis*^*-/-*^ and *Themis*^*+/+*^ mice. We found higher glutamine and glutamate contents in stimulated CD4^+^ T cells from *Themis*^-/-^ mice, pointing towards higher mitochondrial activity (Fig. 4A, B). Higher mitochondrial activity suggests more OXPHOS rather than aerobic glycolysis in *Themis*^*-/-*^ CD4^+^ T cells. T cells with more aerobic glycolysis have mitochondria with a punctate morphology, whereas T cells with more OXPHOS have mitochondria with a tubular morphology.^30^ We tested whether mitochondria from *Themis*^*-/-*^ CD4^+^ T cells were of the punctate type or the tubular type. We observed that even upon activation, *Themis*^*-/-*^ CD4^+^ T cells had equal proportions of mitochondria with a tubular or punctate morphology compared to those of *Themis*^*+/+*^ CD4^+^ T cells, which had mitochondria with a mostly punctate morphology (Fig. 4C). This indicates that *Themis*^*-/-*^ CD4^+^ T cells undergo more OXPHOS than *Themis*^*+/+*^ CD4^+^ T cells, which shift to aerobic glycolysis upon TCR stimulation.

### Themis-deficient CD4^+^ T cells have defective mTOR upregulation

**Fig. 5:**
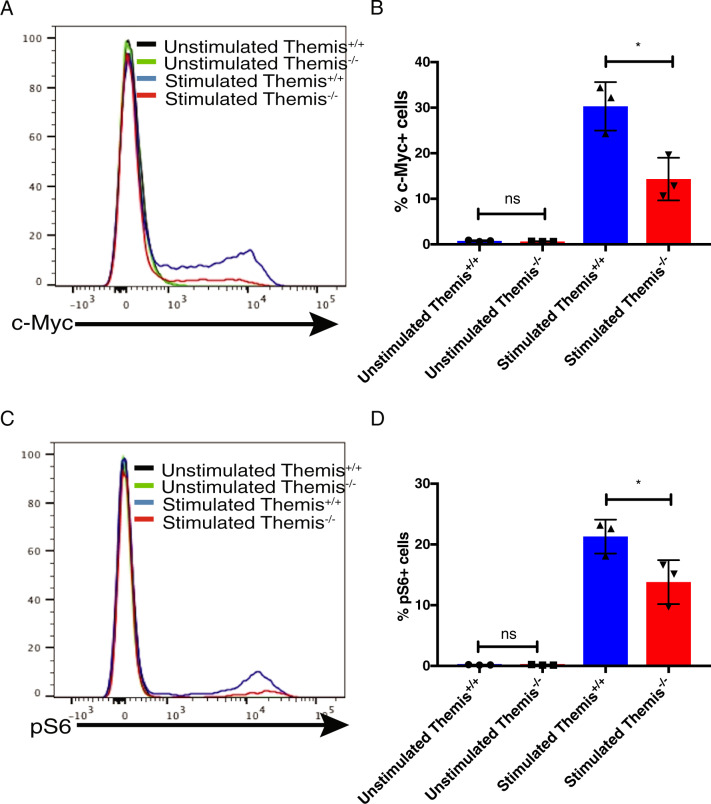
Defective mTOR upregulation in *Themis*^*-/-*^ CD4^+^ T cells upon TCR stimulation. **A** Histogram showing c-Myc expression in stimulated and unstimulated CD4^+^ T cells from *Themis*^*-/-*^ and *Themis*^*+/+*^ mice. **B** Proportions of c-Myc^+^CD4^+^ T cells from *Themis*^*-/-*^ and *Themis*^*+/+*^ mice, with and without TCR stimulation. **C** Histogram showing pS6 expression in stimulated and unstimulated CD4^+^ T cells from *Themis*^*-/-*^ and *Themis*^*+/+*^ mice. **D** Proportions of pS6^+^CD4^+^ T cells from *Themis*^*-/-*^ and *Themis*^*+/+*^ mice, with and without TCR stimulation. Data are representative of three individual experiments with *n* = 3 technical replicates per genotype per experiment. ^ns^not significant, *p < 0.5 as determined by Student’s *t* test. All error bars represent SDs

TCR stimulation and activation leads to activation of the kinase mTOR, which in turn activates c-Myc.^8^ Activation of c-Myc is important for cellular expansion, proliferation and other effector functions.^11,31^ A good measure of mTOR activity is the phosphorylation of its substrate p70S6-kinase, more commonly known as pS6, which indirectly reflects translation in the cell.^32–34^ Since we observed defective upregulation of the IR and glucose transporter after activation in Themis-deficient CD4^+^ T cells (Fig. 3), we hypothesized that there might be differences in the expression of other genes downstream of TCR signaling that would have an effect on T cell effector functions. Upon TCR activation, quiescent T cells grow and increase in size for the first ~24 h and then start to divide every 4–6 h. Since c-Myc and pS6 are responsible for the translation and production of enzymes required for proliferation and effector functions,^11^ we tested their expression at 24 h post-stimulation. We used naïve CD4^+^ Foxp3^-^ T cells sorted from *Themis*^*-/-*^ and *Themis*^*+/+*^ mice and stimulated them with anti-CD3/CD28 for 24 h. These cells were then stained for c-Myc and pS6 and analyzed by flow cytometry. We observed that the proportion of CD4^+^ T cells that were positive for c-Myc and pS6 upon TCR stimulation was lower in *Themis*^*-/-*^ mice than in *Themis*^*+/+*^ mice (Fig. 5). This suggests weaker effector function of *Themis*^*-/-*^ CD4^+^ T cells, possibly explaining the reduced cytokine production we observed in the colitis model (Fig. 2F), since a correlation between effector function, namely, IFNγ production, and c-Myc expression has been shown in previous studies.^25^ The lower frequency of pS6-expressing cells in *Themis*^*-/-*^ CD4^+^ T cells indicates reduced translation of downstream genes, some of which might be needed for T cell effector functions.^34,35^

### NFAT regulates *IR* expression in activated CD4^+^ T cells

**Fig. 6:**
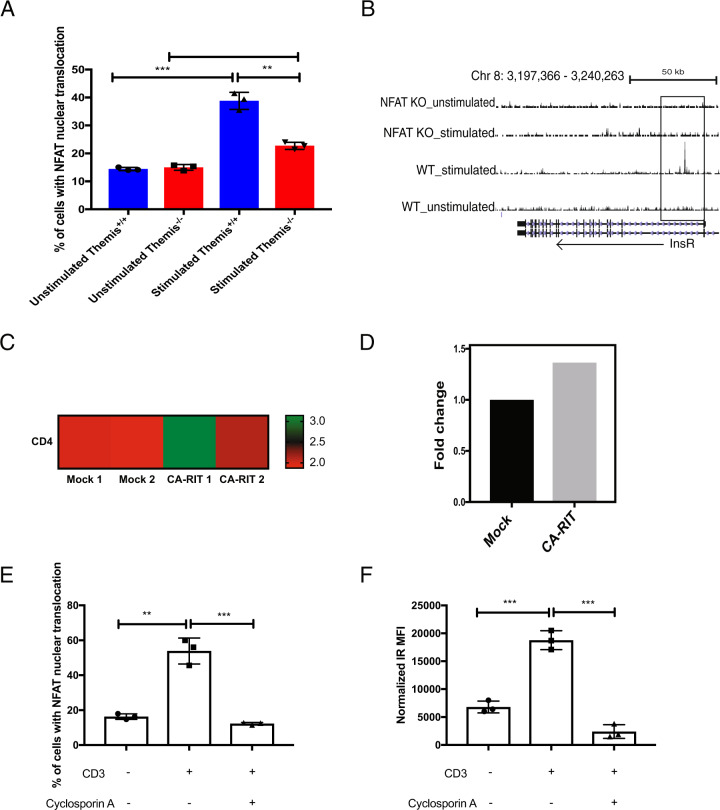
NFAT regulates *IR* gene expression. **A** Proportions of unstimulated and stimulated CD4^+^ T cells from *Themis*^*-/-*^ and *Themis*^*+/+*^ mice with nuclear translocation of NFAT (*n* = 3 technical replicates per genotype). **B** ChIP-Seq data (data-mined from ref. ^37^) showing NFAT binding at exon 1 of *IR* in stimulated T cells. **C** Heatmap of RNA-Seq data (data-mined from ref. ^37^) showing the *IR* expression profile between CD4^+^ T cells from NFAT-WT and NFAT-CA-RIT mice. **D** Fold-change values of *IR* expression differences between CD4^+^ T cells from NFAT-WT and NFAT-CA-RIT mice (data-mined from ref. ^37^). **E** Proportions of unstimulated, stimulated and stimulated in the presence of cyclosporin A, CD4^+^ T cells from *Themis*^*+/+*^ mice with nuclear translocation of NFAT (*n* = 3 technical replicates per genotype). **F** IR expression on unstimulated, stimulated and stimulated in the presence of cyclosporin A, CD4^+^ T cells from *Themis*^*+/+*^ mice (*n* = 3 technical replicates per genotype). The data from (**A**, **E** and **F**) are representative of three individual experiments. **p < 0.01, ***p < 0.001, as determined by Student’s *t* test. All error bars represent SDs

The inability of CD4^+^ T cells from *Themis*^-/-^ mice to upregulate IR suggests that there might be direct or indirect interactions between Themis and IR through their common binding partners, such as Grb2. To test this hypothesis, we immunoprecipitated IR from CTLs and immunoblotted for Themis, IR, GAPDH and Grb2. We confirmed that Grb2 binds to both Themis and IR, but we did not observe any interactions between Themis and IR (supplementary Fig. 2). We therefore sought to examine whether there might be an indirect connection between Themis and IR expression. Previous research has shown that Themis modulates early TCR signaling.^15,21^ TCR signaling results in the nuclear translocation of transcription factors, such as NFAT, which regulate transcription programs for T cell activation and effector functions, such as IL-2 production.^14^ Since we observed reduced IL-2 production in *Themis*^*-/-*^ CD4^+^ T cells (Fig. 1C), we investigated the nuclear translocation of NFAT using imaging flow cytometry.^36^ Naïve CD4^+^ Foxp3^-^ T cells sorted from *Themis*^*+/+*^ and *Themis*^*-/-*^ mice were left unstimulated or stimulated with anti-CD3/CD28 for 3 h. We observed that after stimulation, a smaller proportion of *Themis*^-/-^ CD4^+^ T cells than *Themis*^*+/+*^ CD4^+^ T cells showed NFAT localization to the nucleus (Fig. 6A), suggesting reduced NFAT transcriptional activity in Themis-deficient CD4^+^ T cells. It was therefore important to determine whether *IR* is one of the genes that is regulated by NFAT in activated CD4^+^ T cells. To this end, we analyzed published chromatin immunoprecipitation-sequencing (ChIP-seq) data, which reported ChIP data for NFAT from stimulated and unstimulated wild-type and NFAT-deficient T cells.^37^ This allowed us to look at the potential binding sites of NFAT molecules in the genome. The data showed strong binding of NFAT at exon 1 of *IR* (Fig. 6B), which suggests that NFAT regulates *IR* gene expression upon T cell activation. To further validate this, we examined RNA-seq data from wild-type versus NFAT-CA-RIT CD4^+^ T cells.^37^ CA-RIT cells have constitutive NFAT nuclear localization. The RNA-seq data show that constitutive expression of NFAT in the nucleus led to higher expression of *IR* (Fig. 6C, D), which demonstrates regulation of *IR* gene expression by NFAT. To further confirm this finding, we used cyclosporin A, a known NFAT inhibitor^38^ and tested its effect on IR expression. The addition of cyclosporin A to T cell cultures led to reduced translocation of NFAT to the nucleus and a simultaneous reduction in IR expression on the cell surface (Fig. 6E, F), thus providing further evidence of the regulation of IR expression by NFAT. Therefore, reduced NFAT nuclear translocation in response to TCR signaling in CD4^+^ T cells leads to a decrease in metabolic signaling and effector function in CD4^*+*^ T cells.

## Discussion

Themis is a T cell lineage-specific protein that regulates T cell selection in the thymus by setting the threshold for positive and negative selection.^15–17,21^ Here, we analyzed the activation and metabolic defects of Themis-deficient CD4^+^ T cells. *Themis*^*-/-*^ CD4^+^ T cells showed reduced proliferation and lower IL-2 production in vitro than *Themis*^*+/+*^ CD4^+^ T cells. They also showed lower inflammatory potential in an adoptive transfer model of colitis in vivo. These results demonstrate that defective TCR signaling in CD4^+^ T cells due to the absence of Themis effectively diminishes their effector functions. Genes involved in carbohydrate metabolism were notably downregulated in Themis-deficient developing thymocytes, which also showed signs of oxidative stress, which may indicate similar metabolic stress during positive selection as observed in mature T cells from selection in *Themis*^-/-^ mice.^17^

Effective TCR signaling and metabolic signaling are both important for proper T cell activation and immune response to antigens. Therefore, defects in either of these factors can incapacitate an effective T cell response. Since we observed defective effector functions in Themis-deficient CD4^+^ T cells, we wanted to examine whether TCR signaling defects in these cells would also lead to defects in metabolic signaling. Glucose is the main energy source for T cells upon activation. T cells take up glucose through the action of the glucose transporter molecule GLUT1^2^ and process it via aerobic glycolysis during activation.^1^ The translocation of glucose transporters to the membrane is regulated by IR signaling, which shares common molecules and pathways with TCR signaling. Grb2 and Shp2 are shared molecules, and both TCR and IR signaling lead to the activation of similar pathways, such as Erk/MAPK and mTOR.^39^ To test our hypothesis, we analyzed the upregulation of IR on CD4^+^ T cells upon activation, which would be required to increase insulin signaling and GLUT1 expression. We observed that peripheral naïve CD4^+^ T cells from *Themis*^*-/-*^ mice were not able to upregulate IR expression upon TCR stimulation, at either the mRNA or protein level.

Interestingly, we detected IR expression even on naïve T cells, contradicting previous findings that IR is expressed in T cells only upon activation and not in their naïve quiescent state.^40^ These previous studies had used human samples and other techniques, which might not be as sensitive as those used here. We validated our results using an insulin binding assay, finding that in unstimulated ex vivo cells, the ratio of insulin binding between CD4^+^ T cells from *Themis*^*-/-*^ and *Themis*^*+/+*^ mice was ~1, suggesting equal amounts of IR expression. However, once activated, compared to those from *Themis*^*-/-*^ mice, CD4^+^ T cells from *Themis*^*+/+*^ mice upregulated their insulin binding by two-fold, suggesting a reduced ability to upregulate IR in the absence of Themis. These data suggested IR signaling deficiencies in Themis-deficient mice.

Since IR was not upregulated, indicating that Themis-deficient CD4^+^ T cells would not be able to transduce IR signals to allow glucose uptake, we checked whether glucose uptake in Themis-deficient CD4^+^ T cells was affected. *GLUT1* expression was increased approximately 12-fold in Themis-sufficient cells but only doubled in Themis-deficient cells. After testing glucose uptake, we found that upon TCR stimulation, CD4^+^ T cells from *Themis*^*-/-*^ mice could take up only half the amount of glucose taken up by *Themis*^*+/+*^ cells. These data indicate that there are problems with signal transduction through IR in CD4^+^ T cells from *Themis*^*-/-*^ mice and that Themis-deficient T cells are insulin resistant since they do not respond to the action of insulin.

Mitochondrial function in energy production relies upon glucose uptake and production of pyruvate through glycolysis. It has been shown that under activating conditions, T cells shift from OXPHOS to aerobic glycolysis to meet the metabolic needs of the effector cells.^41^ OXPHOS and aerobic glycolysis have been linked to different mitochondrial morphologies.^30^ We observed that *Themis*^*-/-*^ CD4^+^ T cells had a higher proportion of OXPHOS ‘tubular’-type mitochondria than aerobic glycolysis ‘punctate’-type mitochondria. *Themis*^*-/-*^ CD4^+^ T cells also had higher intracellular concentrations of glutamine and glutamate than *Themis*^*+/+*^ CD4^+^ T cells, which also points toward more OXPHOS than aerobic glycolysis in *Themis*^*-/-*^ CD4^+^ T cells. This suggests defective metabolic reprogramming upon activation.

The signaling molecules c-Myc and pS6 are downstream of mTOR activation by both IR and TCR signaling.^8^ We observed that compared to that of CD4^+^ T cells from *Themis*^+/+^ mice, a lower percentage of CD4^+^ T cells from *Themis*^*-/-*^ mice upregulated c-Myc and pS6. This fits with our effector function and IR signaling data indicating that Themis-deficient CD4^+^ T cells have problems transducing signals from IR and TCR; thus, a lower percentage of these cells upregulate c-Myc and pS6. This shows that both IR and TCR signaling are crucial for efficient metabolic activation of T cells.

Recent work showed that IR is critical for modulating the immune response and that IR-deficient T cells have defective effector functions,^5^ similar to what we have observed in *Themis*^*-/-*^ CD4^+^ T cells. We did not observe any interactions between IR and Themis, although we found that Grb2 and GAPDH interact with IR, as expected.^3^ This suggested that there might be an indirect link between Themis and IR, possibly through TCR signaling. Themis is involved in TCR signaling, and TCR signaling leads to the nuclear localization of transcription factors such as NFAT, which regulate the expression profile of activated T cells. We discovered that reduced TCR signaling due to the absence of Themis led to a reduced proportion of Themis-deficient CD4^+^ T cells with nuclear translocation of NFAT. We analyzed a previously published report on the NFAT gene regulation landscape^37^ and found that NFAT binds to the *IR* gene, suggesting that NFAT might control *IR* expression downstream of TCR signaling in CD4^+^ T cells. To verify this, we analyzed RNA-seq data from NFAT-WT and NFAT-CA-RIT cells, the latter of which have constitutive nuclear localization of NFAT.^37^ We observed higher expression of *IR* in NFAT-CA-RIT cells, suggesting that NFAT nuclear localization controls *IR* expression in activated CD4^+^ T cells. To provide more direct evidence for this, we used cyclosporin A, a known NFAT inhibitor,^38^ to test whether this would affect IR expression. We observed reduced IR expression in T cells cultured with cyclosporin A, thus showing that NFAT translocation to the nucleus is necessary for IR expression. This explains how TCR signaling defects in *Themis*^*-/-*^ CD4^+^ T cells lead to IR signaling defects, which in turn lead to reduced glucose uptake, mitochondrial dysfunction and defective mTOR upregulation, culminating in poor effector functions.

The absence of Themis in T cells leads to signal transduction differences compared to Themis-sufficient T cells.^15–24^ Our research shows how such differences in signal transduction lead to an altered metabolic landscape via the regulation of IR expression by NFAT translocation to the nucleus, ultimately affecting the T cell’s potential to proliferate, produce cytokines and cause inflammation. Future investigation of such internal control circuits during T cell responses in infection models and cancer immunotherapy, where T cells are exhausted and might not be perceiving the TCR signal at its full strength, would provide insights into ways to engineer these T cells to yield better responses.

## Materials and methods

### Mice

*Themis*^-/-^*Foxp3-GFP*, *Themis*^*+/+*^*Foxp3-GFP*, *Rag1*^*-/-*^ and OT-I mice on the C57BL/6 background were bred in our restricted flora (RF) facilities at Comparative Medicine, NUS. Mice were treated under Institutional Animal Care and Use Committee-approved guidelines in accordance with approved protocols.

### Cell sorting

Mice were euthanized and dissected. Lymph nodes from several mice of the same genotype were excised and pooled together to prepare the samples for sorting. These were then mashed through a 70 μm cell strainer into 5 ml of cRPMI. The resulting cell suspensions were then centrifuged at 1200 rpm for 5 min at 4 °C. The resulting cell pellet was surface stained by resuspension in 0.5 ml of cRPMI per mouse containing fluorescently conjugated antibodies at a 1:500 dilution, followed by incubation at 4 °C for 30 min on a shaker. They were then washed with cRPMI and resuspended in 0.5 ml of cRPMI per mouse for sorting. The cells were sorted on either a Sy2000 (Sony Corporation, Tokyo, Japan) or Facsfusion (BD Biosciences, California, USA) system.

### TCR stimulation

100 or 500 μL of anti-CD3ε and anti-CD28 per well were coated onto a 96- or 24-well plate at concentrations of 1 μg/ml and 2 μg/ml in PBS, respectively, and incubated overnight at 4 °C. The wells were washed with PBS for 10 min before seeding the cells for stimulation.

### IL-2 production

A total of 1 × 10^5^ sorted CD4^+^ Foxp3^-^ CD44^lo^ T cells were added to a 96-well plate with either wells precoated with anti-CD3ε and anti-CD28 or empty wells. After 24 h, the cells were centrifuged to collect the supernatant. An IL-2 ELISA kit (eBiosciences, California, USA) was used to measure the amount of IL-2 in the supernatant.

### CTV labeling

For proliferation analysis, cells were labeled with CTV (Life Technologies, California, USA). Cells were centrifuged and resuspended in PBS at a concentration of 2 × 10^6^/ml. Cell Trace Violet was then added to the cell suspension at a concentration of 5 μM. The cells were vortexed immediately and incubated at 37 °C for 10 min while vortexing every 5 min. After incubation, medium was added to quench the reaction at 5 times the original staining volume and further incubated for 5 min at 37 °C. The cells were then centrifuged at 500 g for 5 min at 4 °C. Live cells were counted and then used for subsequent experiments.

### Flow cytometry

For surface staining, cell pellets were resuspended in 100 μl of PBS with 0.5% BSA (FWB: FACS wash buffer) containing dilutions (1:300) of fluorophore-conjugated antibodies specific for cell surface antigens and incubated on ice for 30 min in the dark. The cells were then centrifuged at 1200 rpm at 4 °C for 5 min and resuspended in 300 μl of FWB for flow cytometry analysis. For intracellular staining, the cells from the previous step were resuspended in 0.2 ml of IC fixation buffer (eBiosciences, California, USA) with vortexing, followed by incubation at room temperature for 20 min. The cells were then washed twice with 2 ml of 1X permeabilization buffer (eBiosciences, California, USA), resuspended in 100 μl of FWB containing dilutions (1:250) of fluorophore-conjugated antibodies specific for intracellular antigens and incubated at room temperature for 30 min. The cells were then washed once with 2 ml of 1X permeabilization buffer and then with 2 ml of FWB. The cells were then resuspended in 300 μl of FWB for analysis on a flow cytometer. Then, 25 μl of Count Bright beads (Life Technologies, California, USA) were added to each sample for cell count analysis. The cells were analyzed on a BD LSR Fortessa X-20 flow cytometer (BD Biosciences, California, USA). Flow cytometry data were analyzed using FlowJo software (Treestar, California, USA). All antibodies used for flow cytometry purposes are described in Table 1.

**Table 1:** List of antibodies used for flow cytometry, immunoprecipitation and western blotting

Antigen	Host	Target	Fluorophore	Clone	Company	Catalog no
IFNγ	Rat	Mouse	PE	XMG1.2	eBiosciences	12-7311-82
TNFα	Rat	Mouse	APC	MP6-XT22	eBiosciences	17-7321-82
IL-2	Rat	Mouse	PE-Cy7	JES6-5H4	BioLegend	503832
CD4	Rat	Mouse	BV421	RM4-5	BioLegend	100544
CD4	Rat	Mouse	V450	RM4-5	eBiosciences	48-0042-82
CD4	Rat	Mouse	AF 488	RM4-5	BD Biosciences	557667
CD4	Rat	Mouse	PE	RM4-5	BioLegend	100512
CD4	Rat	Mouse	APC	RM4-5	eBiosciences	17-0042-83
CD4	Rat	Mouse	APC	GK1.5	eBiosciences	17-0041-83
CD8	Rat	Mouse	BUV395	53-6.7	BD Biosciences	563786
CD8	Rat	Mouse	APC	53-6.7	eBiosciences	17-0081-83
CD25	Rat	Mouse	AF 488	7D4	eBiosciences	53-0252-82
CD25	Rat	Mouse	PE	7D4	BD Biosciences	558642
CD25	Rat	Mouse	PE-Cy7	PC61	BioLegend	102016
TCRβ	Rat	Mouse	BV510	H57-597	BD Biosciences	563221
CD44	Rat	Mouse	BV711	IM7	BD Biosciences	563971
Mouse IgG	Goat	Rabbit	AF647	Polyclonal	Invitrogen	A-21245
CD3e	Armenian Hamster	Mouse	Purified	145-2C11	eBiosciences	14-0031-85
CD28	Syrian Hamster	Mouse	Purified	37.51	eBiosciences	16-0281-86
IR (CD220)	Mouse	Mouse	Purified	46/CD220	BD Biosciences	610109
Themis	Rabbit	Mouse	Purified	Polyclonal	Merck	06–1328
Grb2	Mouse	Mouse	Purified	81/GRB2	BD Biosciences	610112
GAPDH	Rabbit	Mouse	Purified	14C10	Cell Signaling	2118
Rabbit IgG	Goat	Rabbit	680LT	Polyclonal	LI-COR Biosciences	926–68021
Mouse IgG	Goat	Mouse	800CW	Polyclonal	LI-COR Biosciences	926–32210
c-Myc	Rabbit	Mouse	Purified	D3N8F	Cell Signaling	13987S
pS6 (S235/236)	Rabbit	Mouse	Purified	D57.2.2E	Cell Signaling	4858S

### T cell adoptive transfer model of colitis

Colitis was induced as described in ref. ^5,27^ Briefly, 5 × 10^5^ sorted CD4^+^ Foxp3^-^ CD44^lo^ T cells per mouse were injected intraperitoneally into 6- to 8-week-old *Rag1*^-/-^ mice. Injected or control animals were monitored for body weight gain or loss every week for a 6-week period. The time of onset of colitis was determined as the time of 20% body weight loss and the appearance of diarrhea. At the end of the follow-up period, the animals were euthanized, and their colons were collected for pathological analysis. The pathology of the colon was characterized as described previously.^27^ The mesenteric (m)LNs and peripheral (p)LNs (pooled cervical, axillary, brachial, and inguinal LNs) from these mice were analyzed for inflammation caused by the transferred T cells. Total T cell numbers and T_reg_ and T_conv_ numbers between the different groups were analyzed. The isolated cells were restimulated by plate-bound anti-CD3/CD28 for 6 h and then analyzed for IFNγ and IL-17α production via intracellular cytokine staining.

### Insulin receptor staining

Cells were stained with surface markers for 30 min on ice. The cells were fixed with 200 μl of IC fixation buffer for 20 min at room temperature and then washed twice with 2 ml of 1X permeabilization buffer. The permeabilized cells were then blocked with 100 μl of 10% goat serum for 30 min at 37 °C and washed with 2 ml of 1X permeabilization buffer. The IR primary antibody was diluted at 1:500 in 1X permeabilization buffer, and 100 μl of the antibody dilution was added to the cell pellet and stained for an hour on ice, followed by a wash with 2 ml of 1X permeabilization buffer. The fluorescently tagged secondary antibody was diluted at 1:2000 in 1X permeabilization buffer, and 100 μl of the antibody dilution was added to the cell pellet and stained for an hour on ice, followed by a wash with 1X permeabilization buffer and a wash with FWB and then analyzed on a flow cytometer. For the negative controls, the same procedure was followed, except for adding the primary antibody. Insulin receptor expression was then obtained after subtracting the MFI of the negative control from the sample.

### Insulin binding assay

A total of 1 × 10^6^ cells were centrifuged in an Eppendorf tube at 500 g for 5 min at 4 °C. The cell pellet was washed with 1 ml of cold PBS at 500 g for 5 min at 4 °C. The cell pellet was then resuspended in 100 μl of KRPH-modified buffer (20 mM HEPES (HyClone, Utah, USA), 5 mM KH_2_PO_4_ (Sigma-Aldrich, Missouri, USA), 1 mM MgSO_4_ (Sigma-Aldrich, Missouri, USA), 1 mM CaCl_2_, 136 mM NaCl (Sigma-Aldrich, Missouri, USA), 4.7 mM KCl (Sigma-Aldrich, Missouri, USA), 10 mM D-glucose, 1% BSA at pH 7.8) containing fluorescent insulin (1:200; Nanocs Inc., New York, USA) and CD4 and CD8 antibodies for surface staining. This cell suspension was then incubated for 90 min at 15 °C in the dark, followed by two washes with 1 ml of cold PBS. The cells were then resuspended in 300 μl of KRPH-modified buffer for analysis on a flow cytometer. For the negative controls, the same procedure was followed, except for the addition of fluorescent insulin. The amount of insulin binding was then calculated after subtracting the MFI of the negative control from the sample.

### qPCR

A total of 5 × 10^5^ cells were stimulated with plate-bound anti-CD3/CD28 for 72 h. RNA was extracted from unstimulated and stimulated cells according to the protocol of the RNeasy spin column (Qiagen, Hilden, Germany). RNA concentrations were measured using an ND-1000 spectrophotometer. The RNA was converted into cDNA with a Biorad iScript cDNA synthesis kit (Biorad, California, USA). The amount of cDNA was normalized among all samples based upon RNA concentrations. The cDNA sequences of the genes were obtained from NCBI GenBank, and Primer 3.0 was used for primer design. qPCR was then conducted on a Light Cycler 96 (Roche, Basel, Switzerland) using the SyBr green detection method according to the manufacturer’s protocol. β-2 microglobulin was used as a housekeeping gene. The analysis was performed by the ΔΔC_t_ method. The list of the primers used for qPCR is as follows:

*IR*, 5′-ATGGGCTTCGGGAGAGGAT-3′ (forward) and 5′-GATGTCCATACCAGGGCACA-3′ (reverse);

*Glut1*, 5′-AGCCCTGCTACAGTGTAT-3′ (forward) and 5′-AGGTCTCGGGTCACATC-3′ (reverse); and

*β-2 microglobulin*, 5′-ACCGGCCTGTATGCTATCCAGAAA-3′ (forward) and 5′-GGTGAATTCAGTGTGAGCCAGGAT-3′ (reverse).

### Glucose uptake assay

The assay was performed according to the manufacturer’s instructions. Briefly, cells were pelleted at 500 g for 5 min at 4 °C and then washed twice with glucose-free medium. 2-NBDG (Cayman Technologies, Ohio, USA) was diluted to a concentration of 150 μg/ml in glucose-free medium. Then, 100 μl of the 2-NBDG dilution was added to the cells and incubated at 37 °C for 30 min in the dark. The cells were washed twice with FWB to remove all the residual 2-NBDG and then analyzed on a flow cytometer.

### Mitochondrial analysis

Glutamine and glutamate intracellular concentrations were calculated using the Glutamine/Glutamate-Glo^TM^ Assay (Promega, Wisconsin, USA) following the manufacturer’s protocol. Mitochondrial morphology analysis was performed as described in ref. ^42^ Briefly, the live cell fraction was isolated from T cells stimulated with plate-bound anti-CD3/CD28 by using lymphocyte separation medium (Corning, Virginia, USA). These cells were then stained in prewarmed serum-free medium containing MitoTracker Green at a concentration of 1 μM for 40 min at 37 °C. The cells were then washed twice with PBS to remove any residual MitoTracker and resuspended in 100 μl of PBS with a 1:1000 dilution of DAPI. The cells were then washed and plated in an 8-well chamber (Nunc, New York, USA) to form a monolayer for microscopic analysis. The samples were then analyzed on an IX83 fluorescence microscope (Olympus, Tokyo, Japan). The images were collected and analyzed on cellSens imaging software (Olympus, Tokyo, Japan).

### mTOR activation analysis

Cells (0.5–1 × 10^6^) were pelleted at 500 g for 5 min at 4 °C. The cells were then fixed by adding 1 ml of 4% PFA while vortexing and incubated at room temperature for 15 min. The cells were then washed with 2 ml FWB, followed by permeabilization of the cells by adding 1 ml of ice-cold 90% methanol while vortexing and incubation on ice for 30 min. The samples were then stored at −20 °C or used for further staining. The cells were then washed twice with 2 ml of FWB to remove all residual methanol. The cell pellet was resuspended in 50 μl of primary antibodies (c-Myc and pS6) diluted 1:100 and stained for an hour at room temperature. The cells were then washed with 2 ml of FWB and resuspended in 50 μl of FWB containing a 1:2000 dilution of fluorescently conjugated secondary antibody and surface markers such as CD4, CD8, and CD25 for 30 min at room temperature in the dark. The cells were then washed and resuspended in FWB for analysis on a flow cytometer.

### CTL culture

Spleens from OT-I mice were excised after euthanasia and mashed through a 70 μm cell strainer into 5 mL of cRPMI. The cell suspension was then centrifuged at 500 *g* for 5 min at 4 °C to obtain the cell pellet. The cell pellet was then resuspended in 2 ml of ACK lysis buffer (150 mM NH_4_Cl (Sigma-Aldrich, Missouri, USA), 10 mM KHCO_3_ (Sigma-Aldrich, Missouri, USA), and 0.1 mM EDTA (Invitrogen, Massachusetts, USA) in Milli-Q water) to lyse the contaminating erythrocytes for 10 min. Lysis was stopped by adding 5 ml of cRPMI to the cell suspension. The samples were then centrifuged at 500 *g* for 5 min at 4 °C to obtain the splenocyte pellet. The splenocyte pellet was resuspended in 40 ml of cRPMI containing IL2 (10 U/ml; PeproTech, New Jersey, USA) and OVA peptide (10 ng/ml; NIH, Maryland, USA) and incubated at 37 °C for 2 days in a T-75 flask. The cell suspension was then centrifuged at 500 g for 5 min at 4 °C. The cell pellet was resuspended in 40 ml of cRPMI containing IL2 (10 U/ml) and incubated for 2 more days at 37 °C in a T-75 flask. The CTLs were used immediately or were cultured for an additional 1–2 days by splitting the cells into two T-75 flasks and adding fresh cRPMI.

### Preparation of cell lysate for immunoprecipitation

Twenty milliliters of CTL culture was spun down at 500 g for 5 min at 4 °C. The supernatant was discarded, and the pellet was washed with 2 ml of PBS at 500 g for 5 min. Finally, this pellet was resuspended in 300 μl of lysis buffer (Brij97 (Sigma-Aldrich, Missouri, USA) for IR and maltoside buffer (Sigma-Aldrich, Missouri, USA) for Themis and transferred to an Eppendorf tube. The samples were then placed on a cold orbital shaker at 750 rpm for an hour at 4 °C, followed by centrifugation for 15 min at 13000 rpm at 4 °C. During lysis, nuclear debris and other insoluble components formed an insoluble pellet that was discarded after transferring the supernatant to a fresh tube. This supernatant was the cell lysate that was used for immunoprecipitation.

### Immunoprecipitation

A total of 30 μl of cell lysate was collected as the whole-cell lysate. Then, 6 μl of 6X SDS reducing agent (Nacalai Tesque Inc., Kyoto, Japan) was added to the whole-cell lysate, followed by heating the sample at 95 °C for 5 min and storing the sample at −80 °C. Next, 15 μl of protein G beads (Invitrogen, Massachusetts, USA) were washed with 200 μl of lysis buffer and then added to the remaining cell lysate for 30 min on a rotary shaker at 4 °C for preclearing to reduce nonspecificity. Then, 6 μg of Ab was added to the precleared cell lysate. This mixture was then incubated overnight at 4 °C on a rotary shaker. The next day, 30 μl of protein G beads were washed twice with 500 μl of lysis buffer and then resuspended in 100 μl of lysis buffer. This sample was then added to the lysate with the Ab. The bead-Ab complex was incubated with the lysate for 2–4 h at 4 °C. The bead-Ag-Ab complex was washed twice with lysis buffer. It was then resuspended in 36 μl of 1X SDS sample buffer, followed by boiling for 5 min at 95 °C in SDS sample buffer to elute the protein.

### Western blotting

Ten microliters of the immunoprecipitated sample and the whole-cell lysate was loaded into 12-well precast SDS gels (Invitrogen, Massachusetts, USA). The gels were then run with 1X MOPS Buffer (Invitrogen, Massachusetts, USA) at 100 V for 20 min and then at 140 V for 90 min. The proteins were then transferred to a methanol-preactivated PVDF membrane (EMD Millipore, Massachusetts, USA) for 1 h at 100 V with 1X transfer buffer. After transfer, the membrane was again activated in methanol for 30 s. The membrane was then blocked in blocking buffer (LI-COR Biosciences, Nebraska, USA) for 1 h at room temperature. Primary antibodies were added at the recommended dilution in blocking buffer, and the membrane was incubated overnight at 4 °C on an orbital shaker. The membrane was washed five times with 1X TBST (20 mM Tris, 150 mM NaCl, 0.2% Tween-20 (Sigma-Aldrich, Missouri, USA) in Milli-Q water) before incubation with another primary antibody or the secondary antibody. The secondary antibody solution was prepared by adding anti-rabbit 680LT and anti-mouse 800CW antibodies to blocking buffer at a 1:4000 dilution. The membrane was washed five times with 1X TBST and air-dried. Processing and analysis of the membrane were carried out on the Odyssey Infrared Imaging System (LI-COR Biosciences, Nebraska, USA). All the antibodies used for immunoprecipitation and western blotting are listed in Table 1.

### Imaging flow cytometry

The assay was performed as described in ref. ^36^ with a few changes. The staining procedure was similar to that used for mTOR pathway markers, except for a 1:500 dilution of NFAT primary antibody. After secondary antibody staining, the cells were centrifuged and resuspended in PBS with a 1:1000 dilution of DAPI for 15 min in the dark. They were then washed with PBS and resuspended in 30 μl of PBS in an Eppendorf tube. They were then analyzed on an Amnis ImageStream X MKII Imaging flow cytometer (Merck Millipore, Darmstadt, Germany). The data were analyzed using IDEAS software (Merck Millipore, Darmstadt, Germany) to calculate nuclear translocation.

### In silico analysis

NFAT ChIP-seq data sets were downloaded from GEO and were analyzed using the UCSC genome browser. RNA-seq data sets were downloaded from GEO, and the RPKM values, calculated based on the read counts using a custom script, were used to generate the heatmap and plot the fold change.

### Statistical analysis

Prism (GraphPad Software, California, USA) and Excel (Microsoft Corporation, Washington, USA) were used for all statistical analysis and graphical representations. Data are presented as the mean ± s.d., and we determined significance by Student’s *t* test. We considered a *p* value of ≤0.05 as statistically significant.

## Supplementary information

Supplementary Figure 1

Supplementary Figure 2

Supplementary Figures

## References

[1] Chang C-H, Pearce EL (2016). Emerging concepts of T cell metabolism as a target of immunotherapy. Nat. Immunol..

[2] Macintyre AN (2014). The Glucose Transporter Glut1 Is Selectively Essential for CD4 T Cell Activation and Effector Function. Cell Metab..

[3] Saltiel AR, Kahn CR (2001). Insulin signalling and the regulation of glucose and lipid metabolism. Nature.

[4] Fischer HJ (2017). The Insulin Receptor Plays a Critical Role in T Cell Function and Adaptive Immunity. J. Immunol..

[5] Tsai S (2018). Insulin Receptor-Mediated Stimulation Boosts T Cell Immunity during Inflammation and Infection. Cell Metab..

[6] Buck MD, O’Sullivan D, Pearce EL (2015). T cell metabolism drives immunity. J. Exp. Med..

[7] O’Sullivan D, Pearce EL (2015). Targeting T cell metabolism for therapy. Trends Immunol..

[8] Waickman AT, Powell JD (2012). mTOR, metabolism, and the regulation of T-cell differentiation and function. Immunol. Rev..

[9] Michalek RD (2011). Cutting edge: distinct glycolytic and lipid oxidative metabolic programs are essential for effector and regulatory CD4+ T cell subsets. J. Immunol..

[10] Doedens AL (2013). Hypoxia-inducible factors enhance the effector responses of CD8(+) T cells to persistent antigen. Nat. Immunol..

[11] Wang R (2011). The Transcription Factor Myc Controls Metabolic Reprogramming upon T Lymphocyte Activation. Immunity.

[12] Nakaya M (2014). Inflammatory T Cell Responses Rely on Amino Acid Transporter ASCT2 Facilitation of Glutamine Uptake and mTORC1 Kinase Activation. Immunity.

[13] Sena LA (2013). Mitochondria Are Required for Antigen-Specific T Cell Activation through Reactive Oxygen Species Signaling. Immunity.

[14] Macián F, López-Rodríguez C, Rao A (2001). Partners in transcription: NFAT and AP-1. Oncogene.

[15] Fu G (2009). Themis controls thymocyte selection through regulation of T cell antigen receptor–mediated signaling. Nat. Immunol..

[16] Lesourne R (2009). Themis, a T cell-specific protein important for late thymocyte development. Nat. Immunol..

[17] Johnson AL (2009). Themis is a member of a new metazoan gene family and is required for the completion of thymocyte positive selection. Nat. Immunol..

[18] Patrick MS (2009). Gasp, a Grb2-associating protein, is critical for positive selection of thymocytes. Proc. Natl Acad. Sci. U. S. A..

[19] Gascoigne NRJ, Rybakin V, Acuto O, Brzostek J (2016). TCR Signal Strength and T Cell Development. Annu. Rev. Cell. Dev. Biol..

[20] Brockmeyer C (2011). T Cell Receptor (TCR)-induced Tyrosine Phosphorylation Dynamics Identifies THEMIS as a New TCR Signalosome Component. J. Biol. Chem..

[21] Fu G (2013). Themis sets the signal threshold for positive and negative selection in T-cell development. Nature.

[22] Mehta M (2018). Themis-associated phosphatase activity controls signaling in T cell development. Proc. Natl Acad. Sci. U. S. A..

[23] Choi S (2017). THEMIS enhances TCR signaling and enables positive selection by selective inhibition of the phosphatase SHP-1. Nat. Immunol..

[24] Brzostek J (2020). T cell receptor and cytokine signal integration in CD8+ T cells is mediated by the protein Themis. Nat. Immunol..

[25] Preston GC (2015). Single cell tuning of Myc expression by antigen receptor signal strength and interleukin-2 in T lymphocytes. Embo. J..

[26] Hawse WF, Cattley RT, Wendell SG (2019). Cutting Edge: TCR Signal Strength Regulates Acetyl-CoA Metabolism via AKT. J. Immunol..

[27] Ostanin DV (2009). T cell transfer model of chronic colitis: concepts, considerations, and tricks of the trade. Am. J. Physiol. Gastrointest. Liver. Physiol..

[28] Sinclair LV (2013). Control of amino-acid transport by antigen receptors coordinates the metabolic reprogramming essential for T cell differentiation. Nat. Immunol..

[29] Blagih J (2015). The energy sensor AMPK regulates T cell metabolic adaptation and effector responses in vivo. Immunity.

[30] Buck MD (2016). Mitochondrial Dynamics Controls T Cell Fate through Metabolic Programming. Cell.

[31] MacIver NJ, Michalek RD, Rathmell JC (2013). Metabolic Regulation of T Lymphocytes. Annu. Rev. Immunol..

[32] Magnuson B, Ekim B, Fingar DC (2012). Regulation and function of ribosomal protein S6 kinase (S6K) within mTOR signalling networks. Biochem. J..

[33] Csibi A (2014). The mTORC1/S6K1 Pathway Regulates Glutamine Metabolism through the eIF4B- Dependent Control of c-Myc Translation. Curr. Biol..

[34] Sasaki CY (2016). p(^70^S^6^K^1^) in the TORC1 pathway is essential for the differentiation of Th17 Cells, but not Th1, Th2, or Treg cells in mice. Eur. J. Immunol..

[35] Düvel K (2010). Activation of a Metabolic Gene Regulatory Network Downstream of mTOR Complex 1. Mol. Cell..

[36] Maguire O, Tornatore KM, O’Loughlin KL, Venuto RC, Minderman H (2013). Nuclear translocation of nuclear factor of activated T cells (NFAT) as a quantitative pharmacodynamic parameter for tacrolimus. Cytom. A..

[37] Martinez GJ (2015). The transcription factor NFAT promotes exhaustion of activated CD8^+^ T cells. Immunity.

[38] Brandt C, Pavlovic V, Radbruch A, Worm M, Baumgrass R (2009). Low-dose cyclosporine A therapy increases the regulatory T cell population in patients with atopic dermatitis. Allergy.

[39] Samelson LE (2002). Signal Transduction Mediated by the T Cell Antigen Receptor: The Role of Adapter Proteins. Annu. Rev. Immunol..

[40] Stentz FB, Kitabchi AE (2004). Transcriptome and proteome expression in activated human CD4 and CD8 T-lymphocytes. Biochem. Biophys. Res. Commun..

[41] Chang C-H (2013). Posttranscriptional control of T cell effector function by aerobic glycolysis. Cell.

[42] Gautam N, Sankaran S, Yason JA, Tan KSW, Gascoigne NRJ (2018). A high content imaging flow cytometry approach to study mitochondria in T cells: MitoTracker Green FM dye concentration optimization. Methods.

