# Supplementary figures and images for: Themis regulates metabolic signaling and effector functions in CD4+ T cells by controlling NFAT nuclear translocation
Source: Cell Mol Immunol. 2020 Nov 11;18(9):2249–61. doi: 10.1038/s41423-020-00578-4 (PMC8429700; doi:10.1038/s41423-020-00578-4)

Themis<sup>+/+</sup>

Themis<sup>-/-</sup>

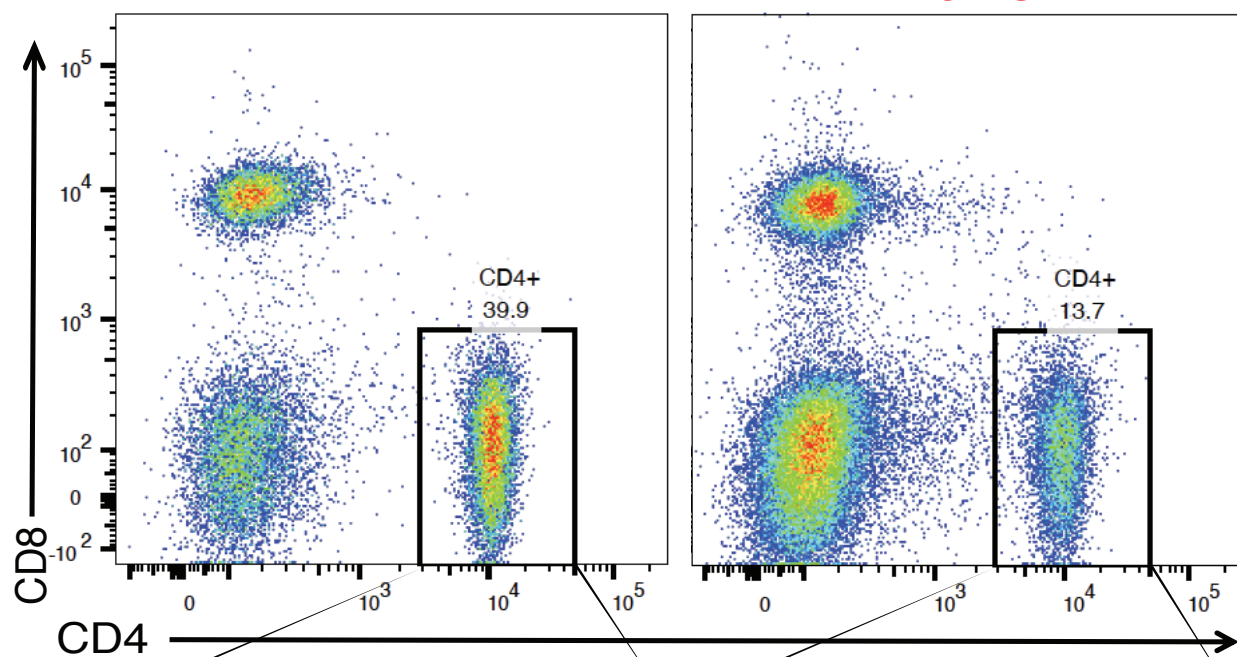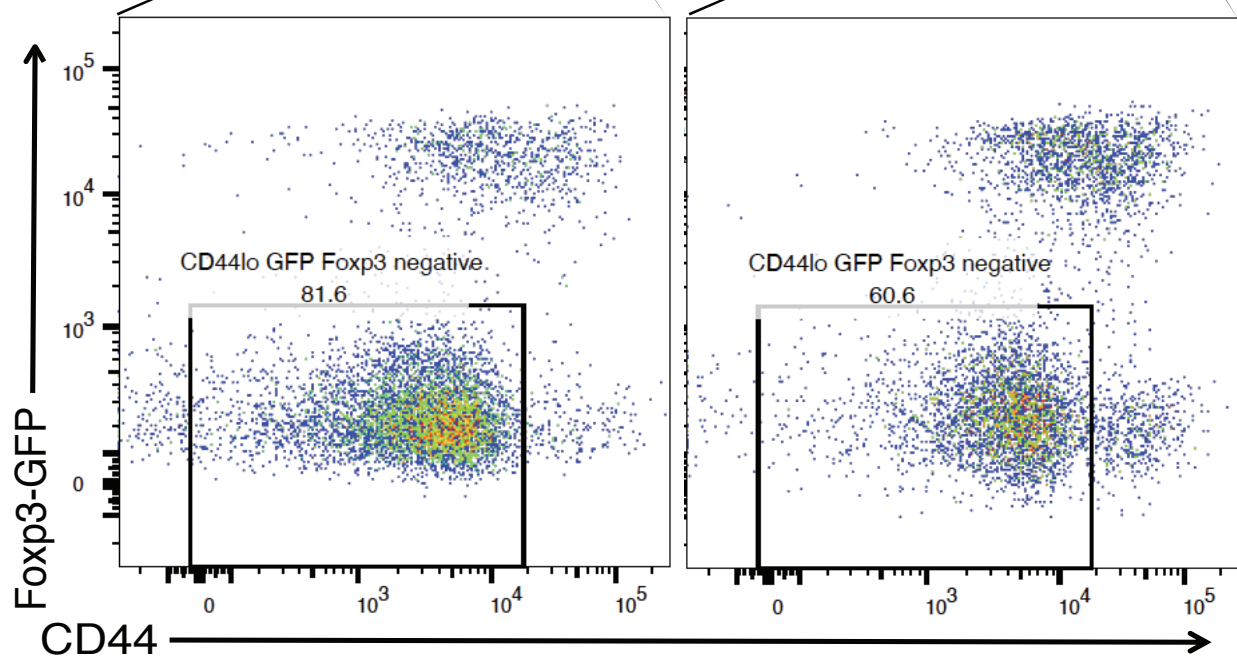

Supplement: Supplementary file 1 — Supplementary Figure 1 [file 41423_2020_578_MOESM1_ESM.pdf]
