# Supplementary material for: Themis regulates metabolic signaling and effector functions in CD4+ T cells by controlling NFAT nuclear translocation
Source: Cell Mol Immunol. 2020 Nov 11;18(9):2249–61. doi: 10.1038/s41423-020-00578-4 (PMC8429700; doi:10.1038/s41423-020-00578-4)
Supplement: Supplementary file 2 — Supplementary Figure 2 [file 41423_2020_578_MOESM2_ESM.pdf]

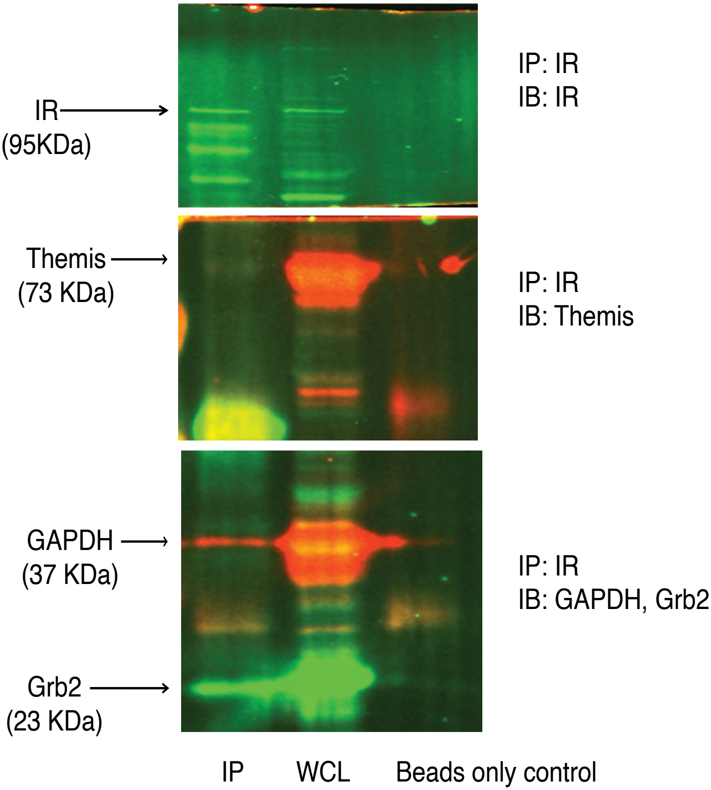
IR
(95KDa)
IP: IR
IB: IR
Themis
(73 KDa)
IP: IR
IB: Themis
GAPDH
(37 KDa)
Grb2
(23 KDa)
IP: IR
IB: GAPDH, Grb2
IP
WCL
Beads only control
