# Supplementary material for: Themis regulates metabolic signaling and effector functions in CD4+ T cells by controlling NFAT nuclear translocation
Source: Cell Mol Immunol. 2020 Nov 11;18(9):2249–61. doi: 10.1038/s41423-020-00578-4 (PMC8429700; doi:10.1038/s41423-020-00578-4)
Supplement: Supplementary file 3 — Supplementary Figures [file 41423_2020_578_MOESM3_ESM.docx]

**Supplementary Figure 1.** Gating strategy for sorting naïve (Foxp3^-^CD44^lo^) CD4^+^ T cells.

**Supplementary Figure 2.** Immunoprecipitation of IR from CTLs and immunoblotting for IR, Themis, GAPDH and Grb2.
